# Outcomes of COVID-19 adults managed in an outpatient versus hospital setting

**DOI:** 10.1371/journal.pone.0263813

**Published:** 2022-02-14

**Authors:** Ninh T. Nguyen, Justine Chinn, Katherine Kirby, Samuel F. Hohmann, Alpesh Amin

**Affiliations:** 1 From the Department of Surgery, University of California, Irvine Medical Center, Orange, California, United States of America; 2 From the Department of Statistics, University of California, Irvine Medical Center, Orange, California, United States of America; 3 From Vizient, Centers for Advanced Analytics and Informatics and Department of Health Systems Management Rush University, Chicago, Illinois, United States of America; 4 From the Department of Medicine, University of California, Irvine Medical Center, Orange, California, United States of America; Osakidetza Basque Health Service, SPAIN

## Abstract

**Introduction:**

The coronavirus disease 2019 (COVID-19) pandemic continues to spread globally and as of February 4, 2021, there are more than 26 million confirmed cases and more than 440,000 deaths in the United States (US). A top priority of the Centers for Disease Control and Prevention (CDC) is to identify risk factors for severe COVID-19 illness. The objective of this study was to analyze the characteristics and outcomes of COVID-19 adults who were managed in an outpatient setting compared to patients who required hospitalization at US academic centers.

**Methods:**

Using the Vizient clinical database, Discharge records of adults with a diagnosis of COVID-19 between March 1, 2020 and January 31, 2021 were reviewed. Outcome measures included demographics, characteristics, rate of hospitalization, and mortality, and data were analyzed based on inpatient versus outpatient management.

**Results:**

Among COVID-19 adults, 1,360,078 patients were managed in an outpatient setting while 545,635 patients required hospitalization. Compared to hospitalized COVID-19 adults, COVID-19 adults who were managed in an outpatient setting were more likely to be female (56.1% vs 47.5%, p <0.001), white (57.7% vs 54.8%, p <0.001), within younger age group of 18–50 years (p<0.001) and have lower rate of comorbidities. Mortality was significantly lower in outpatient group compared to hospitalized group (0.2% vs 12.2%, respectively, p <0.01%). For outpatient group, mortality increased with increasing age group: 0.02% (52 of 295,112) for patients 18–30 years and 1.2% (1,373 of 117,866) for patients >75 years. The rate of hospitalization was lowest for age group 18–30 years at 10.6% (35,607 of 330,719) and highest for age group >75 years at 56.1% (150,381 of 268,247).

**Conclusion:**

This analysis of US academic centers showed that 28.6% of COVID-19 adults who sought care at one of the hospitals reporting data to the Vizient clinical database required in-patient treatment. The rate of hospitalization in our study was lowest for the youngest age group of 18–30 years and highest for age group >75 years. Beside older age, other factors associated with outpatient management included female gender, white race, and having commercial insurance.

## Introduction

The coronavirus disease 2019 (COVID-19) pandemic continues to spread globally and as of February 4, 2021, there are more than 26 million confirmed cases and more than 440,000 deaths in the United States (US) [1]. One of the top priorities of the Centers for Disease Control and Prevention (CDC) is to identify risk factors for severe COVID-19 illness that can lead to hospitalization [1]. Some of the current known risk factors include older adults and patients with certain underlying medical conditions [1]. In a study of 44,672 confirmed COVID-19 cases in China, Wu and colleague reported the spectrum of disease as mild/moderate in 81%, severe in 14%, and critical in 5% of cases with age being a strong predictor for complications and death [2]. The decision for hospitalization often depends on the clinical presentation, risk factors for severe disease and its progression, and the presence of COVID-19 complications such as pneumonia, respiratory failure, sepsis, or cardiac and kidney injury. The objective of this study was to analyze the characteristics and outcomes of COVID-19 adults who were managed in an outpatient setting compared to patients who required hospitalization at US academic centers.

## Methods

The data for this study were obtained from the Vizient clinical database/resource manager (CDB/RM^™^) which is an administrative, clinical, and financial database of more than 650 academic centers and their affiliates. Approval for the use of the data was obtained from Vizient and from the Institutional Review Board of the University of California, Irvine as exempted status due to lack of patient-level data and absence of any patient-identifying information. Discharge records of adults 18 years or older with a diagnosis of COVID-19 who were managed in an outpatient versus hospital setting between March 1, 2020 and January 31, 2021 were reviewed. COVID-19 patients were identified using International Classification of Disease, Tenth edition code of U07.1. Outpatient management was defined as management in an outpatient setting including emergency room or 23 hours management without conversion to an in-patient hospital admission. Outcome measures included demographics, characteristics, rate of hospitalization, and mortality. Chi-Square Test of Independence and Student’s T-tests were performed to determine if statistically significant associations exist between categorical variables. No post hoc analyses were performed. p-values were not adjusted for multiple comparisons. Significance was set at P<0.05. R version 4.0.3 was used for statistical analysis. Mann-Whitney test was used to compare median length of stay between outpatient and hospitalized patients.

## Results

Among COVID-19 adults, 1,360,078 patients were managed in an outpatient setting while 545,635 patients required hospitalization. The rate of hospitalization for COVID-19 was 28,6% (545,635 of 1,905,713). Characteristics of COVID-19 adults managed in an outpatient vs. hospital setting is depicted in Table 1. Compared to hospitalized COVID-19 adults, COVID-19 adults who were managed in an outpatient setting were more likely to be female (56.1% vs 47.5%, p <0.01), white patients (57.7% vs 54.8%, p <0.001), within younger age group of 18–50 years (p<0.01), and have lower rate of comorbidities. Of the 1,360,078 patients who sought outpatient care, 333,137 (24.5%) were seen in the Emergency Department and not admitted to inpatient care. Outcomes of COVID-19 adults managed in an outpatient vs. hospital setting are depicted in Table 2. Mortality was significantly lower in outpatient group compared to hospitalized group (0.2% vs 12.2%, respectively, p <0.01%).

**Table 1:** Summary of characteristics of COVID-19 patients required management in an outpatient vs. hospital setting.

	Hospitalized (n = 545,635)	Outpatient (n = 1,360,078)	P Values
Sex, No (%)			<0.001
Female	259,269 (47.5)	763,133 (56.1)	
Male	286,324 (52.5)	596,660 (43.9)	
Age group, No. (%)			<0.001
18–30	35,607 (6.5)	295,112 (21.7)	
31–50	101,753 (18.6)	464,876 (34.2)	
51–64	141,501 (25.9)	325,762 (24.0)	
65–74	116,393 (21.3)	156,462 (11.5)	
≥ 75	150,381 (27.6)	117,866 (8.7)	
Race/Ethnicity, No. (%)			<0.001
White	299,081 (54.8)	785,383 (57.7)	
Black	121,331(22.2)	250,355 (18.4)	
Asian	18,553 (3.4)	39,988 (2.9)	
Hispanic*	107,431 (19.7)	258,132 (19.0)	
Other	106,670 (19.5)	284,352 (20.9)	
Payer, No. (%)			<0.001
Commercial	136,182 (25.0)	716,643 (52.7)	
Medicare/Medicaid/State-assisted	386,907 (70.9)	611,203 (44.9)	
Other	22,543 (4.1)	32,232 (2.4)	
Existing comorbidities, No. (%)			
Obesity	156,645 (28.7)	34,929 (2.6)	<0.001
Hypertension	341,525 (62.6)	154,305 (11.3)	<0.001
Diabetes	208,711 (38.3)	115,411 (8.5)	<0.001
Anemia	113,675 (20.8)	5,984 (0.44)	<0.001
Chronic pulmonary disease	118,176 (21.7)	19,201 (1.4)	<0.001
Renal failure	116,063 (21.3)	9,438 (0.69)	<0.001
Congestive heart failure	86,764 (15.9)	18,651 (1.4)	<0.001
Liver disease	28,682 (5.3)	8,371 (0.62)	<0.001
Valvular disease	25,856 (4.7)	163 (0.02)	<0.001
Peripheral vascular disease	23,039 (4.2)	1915 (0.14)	<0.001

^†^ Chi-square tests.

* Including White Hispanic, Black Hispanic, Asian Hispanic, and other Hispanic.

**Table 2:** Outcomes for COVID-19 patients required management in an outpatient vs. hospital setting.

Outcomes	Hospitalized (n = 545,635)	Outpatient (n = 1,360,078)	P Value*
Overall mortality, n (%)	66,507 (12.2)	2,767 (0.20)	<0.01
In-hospital mortality according to sex, n (%)			
Female	26,563 of 259,269 (10.2)	1,198 of 763,133 (0.16)	<0.01
Male	39,935 of 286,324 (13.9)	1,559 of 596,660 (0.26)	<0.01
In-hospital mortality according to age group, n (%)			
18–30	524 of 35,607 (1.5)	52 of 295,112 (0.02)	<0.01
31–50	4,194 of 101,753 (4.1)	263 of 464,876 (0.06)	<0.01
51–64	13,089 of 141,501 (9.3)	510 of 325,762 (0.16)	<0.01
65–74	17,723 of 116,393 (15.2)	569 of 156,462 (0.36)	<0.01
≥ 75	30,977 of 150,381(20.6)	1,373 of 117,866 (1.2)	<0.01
Median length of hospital stay (days)	5 days, IQR 3–10 day	1 day, IQR 1–1 day	<0.0001

* Chi-squared or Student’s t-tests with unequal variance.

%). For outpatient group, mortality increased with increasing age group: 0.02% (52 of 295,112) for patients 18–30 years and 1.2% (1,373 of 117,866) for patients >75 years.

**Fig 1:**
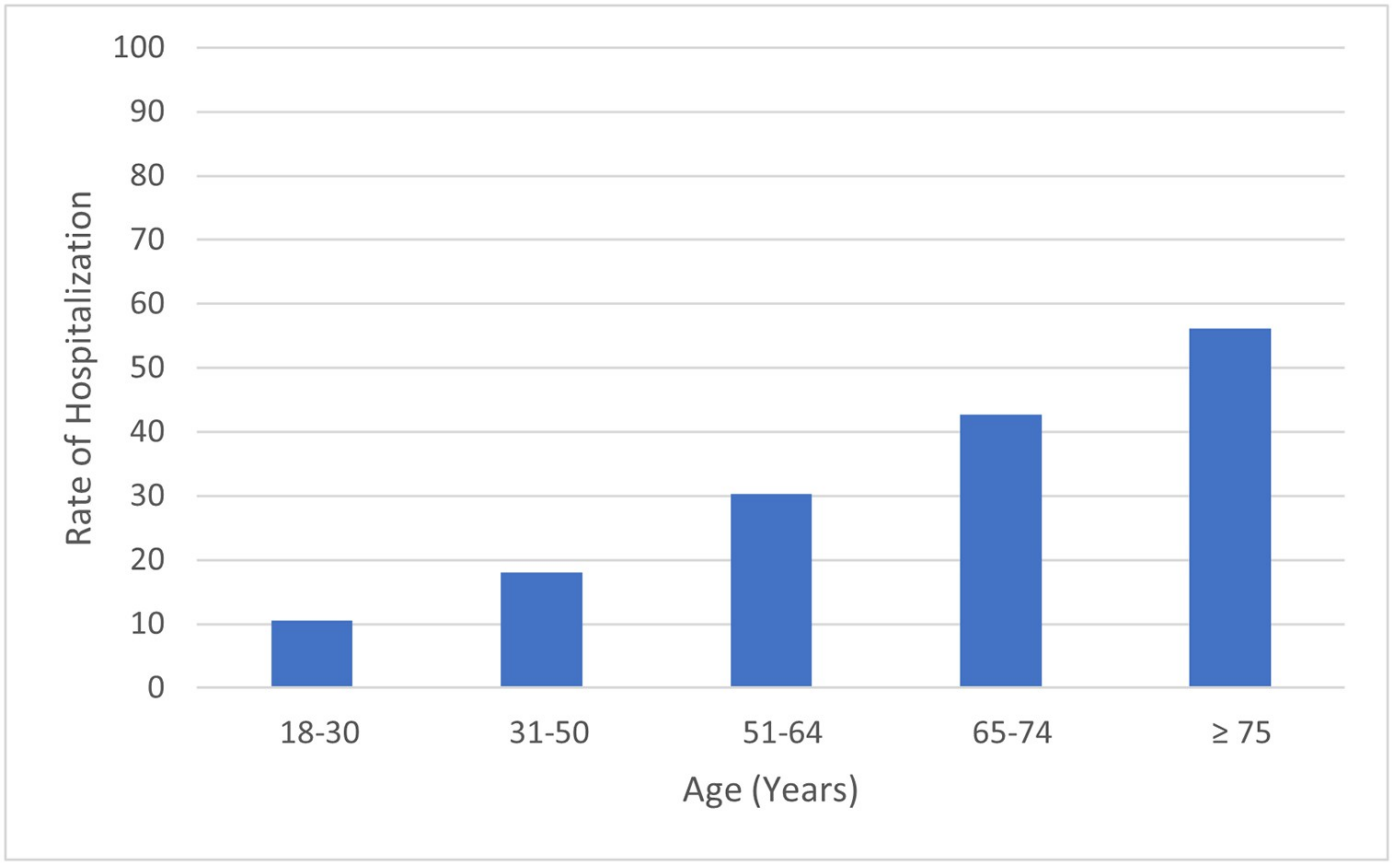
Percentage of COVID-19 adults who sought care at participating Vizient hospitals who received inpatient care according to age group.

**Fig 2:**
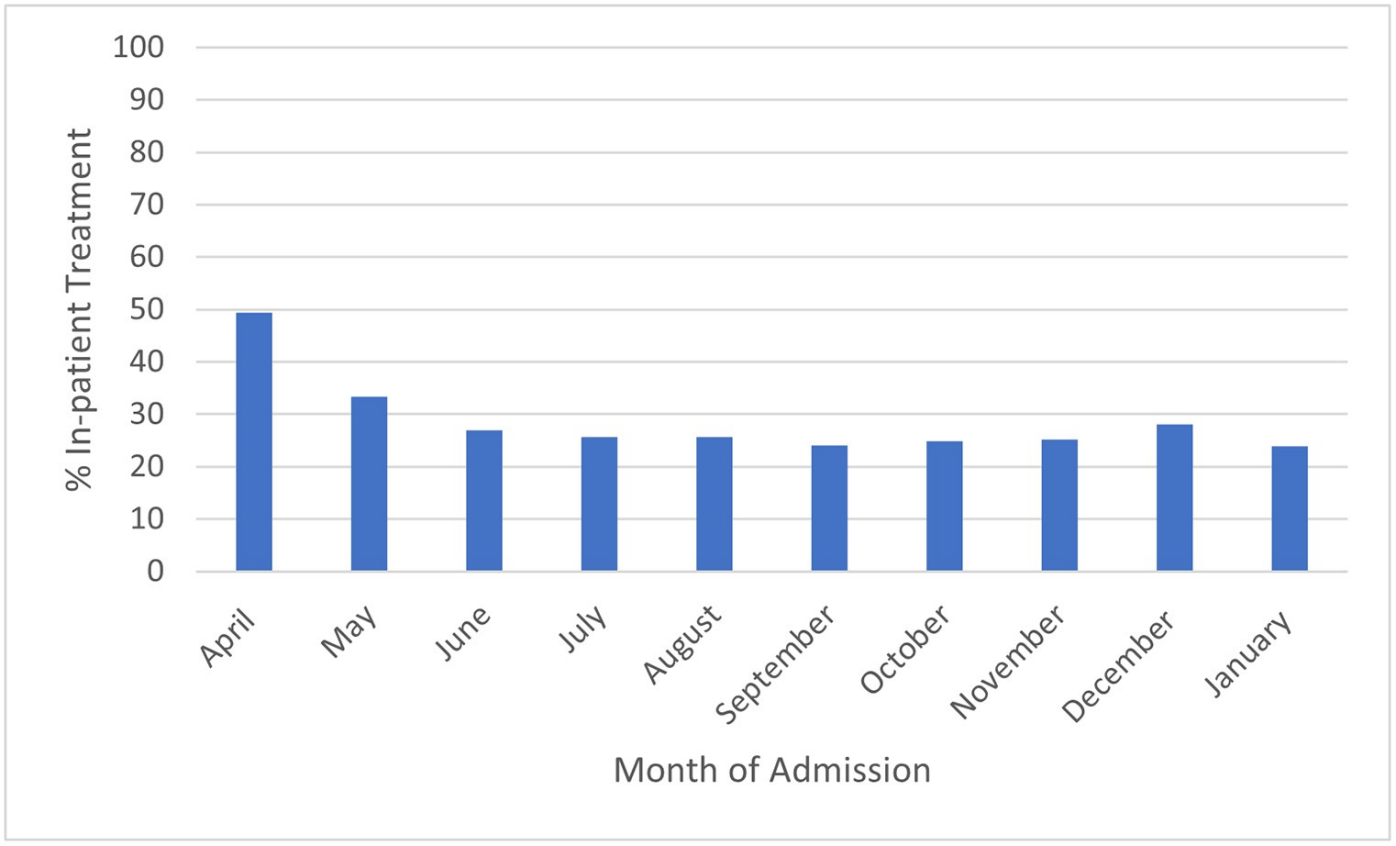
Percentage of COVID-19 adults who sought care at participating Vizient hospitals who received inpatient care according to month of the pandemic from April 2020 through January 2021.

The percentage of COVID-19 adults requiring hospitalization according to age group is depicted in Fig 1. The rate of hospitalization was lowest for age group 18–30 years at 10.6% (35,607 of 330,719) and highest for age group >75 years at 56.1% (150,381 of 268,247). The median length of stay for inpatient care was 5 days (IQR 3–10 days) and for outpatient care was 1 day (IQR 1–1 day). The percentage of COVID-19 adults requiring hospitalization according to the month of the pandemic is depicted in Fig 2.

## Discussion

This analysis of US academic centers showed that 28.6% of patients who sought medical care at one of the hospitals within our database were hospitalized. The reported rate of hospitalization for COVID-19 has been variable ranging between 14.0–39.7% [2–5]. The rate of hospitalization in our study was lowest for the youngest age group of 18–30 years and highest for age group ≥75 years. Beside older age, other factors associated with outpatient management included female gender, white race, and having commercial insurance. Our finding is in concordance with studies showing that hospitalized COVID-19 patients are commonly older, male, and of black race [5, 6]. As expected, the mortality in our study was much higher for hospitalized patients compared to patients managed in an outpatient setting. As described in Fig 2, the percentage of adults with COVID-19 required hospitalization was much higher at the start of the pandemic. We hypothesize that this is likely due to improvements in outpatient treatment of COVID-19. At the start of the pandemic, proven treatments such as steroids and anticoagulation were not widely utilized or proven effective Additionally, treatments such as remdesivir can now be administered in an outpatient infusion center, decreasing the number of people requiring inpatient care [7]. Similarly, more hospitals have engaged the use of self-monitoring at home with pulse oximetry and telehealth rather than inpatient observation. There are several limitations to this retrospective study including misclassification and accuracy of coding and missing data. Input of preexisting comorbidities in the outpatient setting is not as complete as that of the inpatient setting. There could be patients managed in the outpatient setting that also required hospitalization and their data would be present in both groups. Despite these limitations, this study provides data on the high association between increasing age with increasing need for hospitalization and the reduction in the rate of hospitalization as pandemic progressed.

## Abbreviations

CDC: Centers for Disease Control and Prevention
COVID-19: Coronavirus 2019
US: United States
